# A compact platform for the investigation of material dynamics in quasi-isentropic compression to ~ 19 GPa

**DOI:** 10.1038/s41598-021-99479-3

**Published:** 2021-10-19

**Authors:** Yu Lu, Kaiguo Chen, Cheng Cheng, Zhongyu Zhou, Binqiang Luo, Xuemiao Chen, Xuping Zhang, Guiji Wang, Fuli Tan, Jianheng Zhao, Zhuowei Gu, Chengwei Sun

**Affiliations:** 1 Department of Modern Mechanics, University of Science and Technology of China, Hefei, 230027 China; 2 Institute of Fluid Physics, China Academy of Engineering Physics, Mianyang, 621900 China; 3 Department of Physics, National University of Defense Technology, Changsha, 410073 China; 4 Institute of Applied Electronics, China Academy of Engineering Physics, Mianyang, 621900 China

**Keywords:** Applied physics, Condensed-matter physics, Techniques and instrumentation

## Abstract

This paper reports on the development of a magnetically driven high-velocity implosion experiment conducted on the CQ-3 facility, a compact pulsed power generator with a load current of 2.1 MA. The current generates a high Lorentz force between inner and outer liners made from 2024 aluminum. Equally positioned photonic Doppler velocimetry probes record the liner velocities. In experiment CQ3-Shot137, the inner liner imploded with a radial converging velocity of 6.57 km/s while the outer liner expanded at a much lower velocity. One-dimensional magneto-hydrodynamics simulation with proper material models provided curves of velocity versus time that agree well with the experimental measurements. Simulation then shows that the inner liner underwent a shock-less compression to approximately 19 GPa and reached an off-Hugoniot high-pressure state. According to the scaling law that the maximum loading pressure is proportional to the square of the load current amplitude, the results demonstrate that such a compact capacitor bank as CQ-3 has the potential to generate pressure as high as 100 GPa within the inner liner in such an implosion experiment. It is emphasized that the technique described in this paper can be easily replicated at low cost.

## Introduction

The quasi-isentropic compression experiment (ICE)^1^ compresses materials to off-Hugoniot states in which the pressure and temperature are decoupled. In contrast, the material state in an adiabatic shock compression is restricted to the Hugoniot state in which pressure and temperature are coupled. In recent years, the ICE has received interest in the fields of material dynamics^2–6^, condensed matter physics^7–9^, laboratory astrophysics^10^, and inertial fusion^11–13^. There are extensive platforms on which the ICE can be conducted, including the gas gun, pulsed power device, and laser facility. The gas gun can only be used for a planar ICE, whereas a pulsed power or laser facility usually requires heavy investment and an operational team. Examples of such facilities are Z^14^ at the Sandia National Laboratory, Pegasus I/II^15,16^ and Atlas^17,18^ at the Los Alamos National Laboratory, Shiva Star^19,20^ at the Air Force Research Laboratory, magnetic compression generators^21,22^ at the All-Russian Scientific Research Institute of Experimental Physics and Los Alamos National Laboratory, NIF^23^ at the Lawrence Livermore National Laboratory, and OMEGA^24^ at Rochester University. In this paper, we demonstrate ICE via cylindrical implosion at a relatively compact pulsed-power facility, CQ-3 at the Institute of Fluid Physics in China^25^.

Pulsed power devices usually provide great current exceeding 1 MA with a rise time of 10^1^ to 10^3^ ns. This current flows through a load configuration to generate high pressure in the sample. The current rises smoothly from zero to a peak, and it has thus been widely accepted that materials undergo quasi-isentropic compression in such experiments. There are usually two types of load configuration for ICEs conducted on pulsed power devices. The first uses a strip-line sample to realize a plane strain compression state^26,27^. The second uses a cylindrical liner sample to realize a converging implosion state^2,28^. For the first configuration, the peak pressure is much lower than expected because the dynamic inductance due to the two strip electrodes increases rapidly with the distance between the electrodes during loading. In contrast, the cylindrical implosion configuration realizes much higher pressure because of its low and steady dynamic inductance and convergence effect. This configuration uses two cylindrical liners, namely an inner liner and outer liner. The current flows from the inner liner to the outer liner, generating a strong Lorentz force that drives the solid inner liner to a rapid converging velocity and thus generates high pressure within the liner.

The pulsed power devices requiring large investment mentioned above typically realize pressure as high as > 100 GPa via current exceeding 10 MA in ICEs. There are technical and economic difficulties in developing such large facilities. Meanwhile, some laboratories have developed relatively compact pulsed power devices, such as Veloce^29^, Phelix^30^, GEPI^31^, CEPAGE^32^, and the CQ series^25,33^. These facilities usually output current of 1–10 MA and thus have a much lower loading ability. However, they have advantages relating to convenience, low cost, and easy operation. Combining the finite experimental information (such as velocity) with simulation that provides insight into material states (such as pressure and temperature), has become a paradigm for studying material properties under the extreme conditions.

In this paper, we demonstrate implosion quasi-isentropic compression on CQ-3, which is a compact pulsed power device at the Institute of Fluid Physics. A magnetically driven metal liner implosion experiment conducted at high velocity on the CQ-3 device is described. As an integrated experiment, the material model and simulation algorithm can be studied and validated comprehensively. The device parameters of CQ-3 and the details of the experimental assembly are described in Section 2. The experimental results of CQ3-Shot137, namely the liner velocity histories and load current waveform, are presented in Section 3. One-dimensional magnetohydrodynamics (MHD) calculations and data analyses of this shot are presented in Section 4. Section 5 summarizes the results of the study and suggests future work.

## Experimental scheme

### Experimental device and load configuration

**Figure 1:**
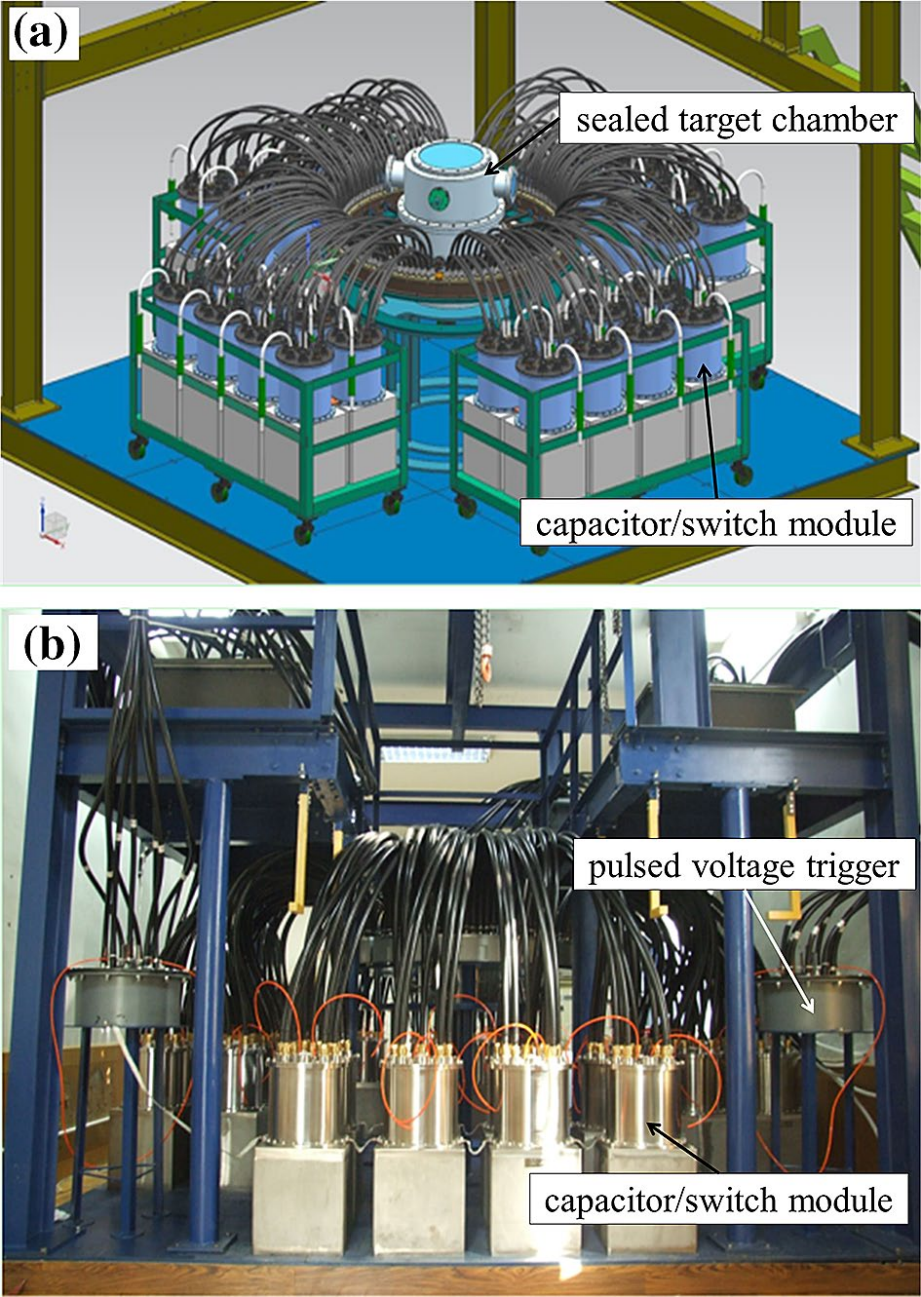
(**a**) Conceptual design and (**b**) photograph of the CQ-3 device.

The CQ-3 device has energy storage of 78 kJ, a charging voltage of 60–90 kV, a maximum short-circuit discharge current of 3 MA, and a rise time (10–90%) of 470 ns. The overall volume of the CQ-3 device is 3.4 m × 3.4 m × 3.0 m. The main components are 32 capacitor/switch modules, four multi-channel triggers, hundreds of low-inductance coaxial cable transmission lines with transition connectors, and a sealed target chamber at the center of the device. The capacitor bank is divided into four groups, all sharing an eight-channel pulsed voltage trigger. The waveform of the discharging current can be adjusted by modifying the action time of each trigger. The dimensions of the target chamber are a diameter of 70 cm and a height of 70 cm, and the vacuum inside the chamber can reach 10^−2^ Pa. The conceptual design and a photograph of the CQ-3 device are shown in Fig. 1.

Compared with other compact devices, CQ-3 has its own unique advantages. Firstly, in contrast with Veloce, GEPI, CEPAGE and CQ-4^33^, it is feasible for CQ-3 to conduct higher vacuum in the target chamber, because the energy transfer mode of CQ-3 is coaxial cables rather than parallel metallic plates. Therefore, it is suitable to carry out some experiments with high sealing requirements or toxic materials on CQ-3. Secondly, besides the strip-line experiment, CQ-3 is also suitable for cylindrical liner experiment since its target chamber is located in the geometric center of the energy storage capacitors, which makes the current evenly distributed in the circumferential direction and the implosion symmetric. For both Phelix and CQ3 devices, which use coaxial cable transmission lines, the difference is that the current rise time of Phelix is about 10 μs, making it suitable for studying the implosion dynamics of larger liner. The current rise time of CQ-3 is less than 500 ns, making it more suitable for small liner experiments with high implosion velocity and compression.

**Figure 2:**
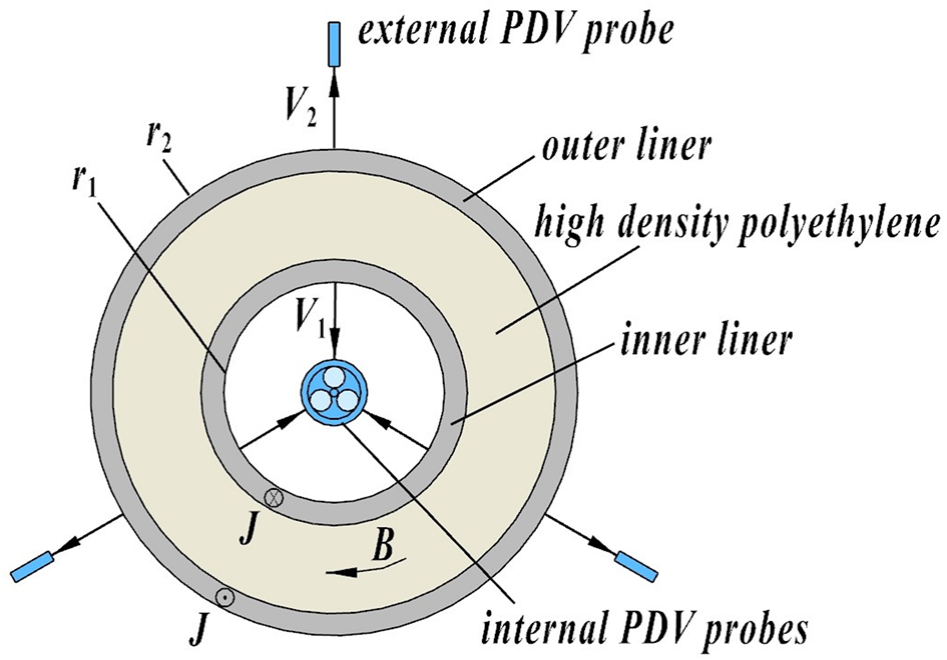
*r–θ* section of the liner configuration.

The cylindrical load configuration comprises two coaxial cylindrical liners (Fig. 2). The lower ends of the inner and outer liners are connected with the cathode and anode flanges respectively. The upper ends are connected through a short-circuit cap to form a series circuit. The space between the two liners is filled with high-density polyethylene as the insulating medium. An axial load current flows through both liners and interacts with the associated azimuthal magnetic field to generate a radial Lorentz force that accelerates the convergent contraction of the inner liner and the outward expansion of the outer liner (respectively referred to as the implosion and expansion below). The material of the inner liner is compressed by ramp waves during its implosion whereas the outer liner withstands a hoop tension.

In experiment CQ3-Shot137, both inner and outer liners were composed of 2024 aluminum. The liners had initial inner radii of 2.485 and 4.990 mm and thicknesses of 515 and 495 μm, respectively. The effective height of the liners was 10 mm, and the surface roughness was approximately 200 nm. Both the short-circuit cap (the upper electrode) and the transmission flanges (the lower electrodes) were made from 304 stainless steel. The surface roughness of the upper and lower electrodes was approximately 3.2 μm. The physical design of the liner configurations was based on the thin-shell model^34^ incorporating circuit equations and one-dimensional MHD modeling, where optimum parameters of the liner and its implosion for achieving higher velocities were considered.

### Configuration layout and diagnostic techniques

**Figure 3:**
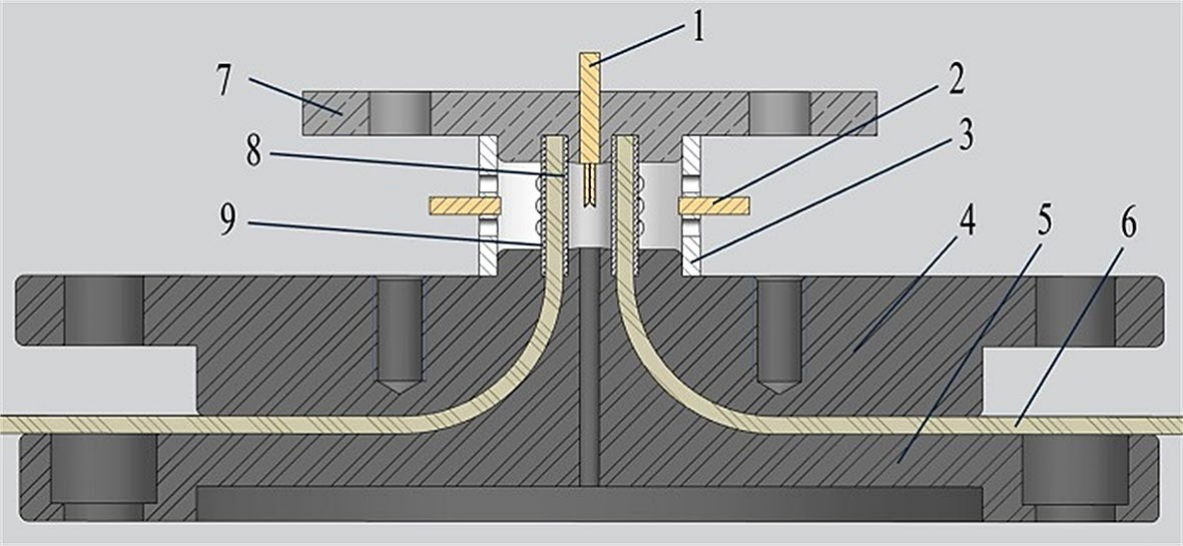
Installation layout of the load configuration in CQ3-Shot137. (1. internal photonic Doppler velocimetry probes 2. external photonic Doppler velocimetry probe 3. probe support 4. anode flange 5. cathode flange 6. high-density polyethylene insulation 7. short-circuit cap 8. inner liner 9. outer liner).

The installation layout of the load configuration (liners) and photonic Doppler velocimetry (PDV) probes on the experimental platform (i.e., the flange plates) is shown in Fig. 3. The liners were accurately positioned relying on the precision machined flanges, and good electrical contacts were ensured in the same way. The experiment with a charging voltage of 80 kV was carried out at ambient temperature and pressure. The load current was measured using the Rogowski belt mounted on the cathode flange.

**Figure 4:**
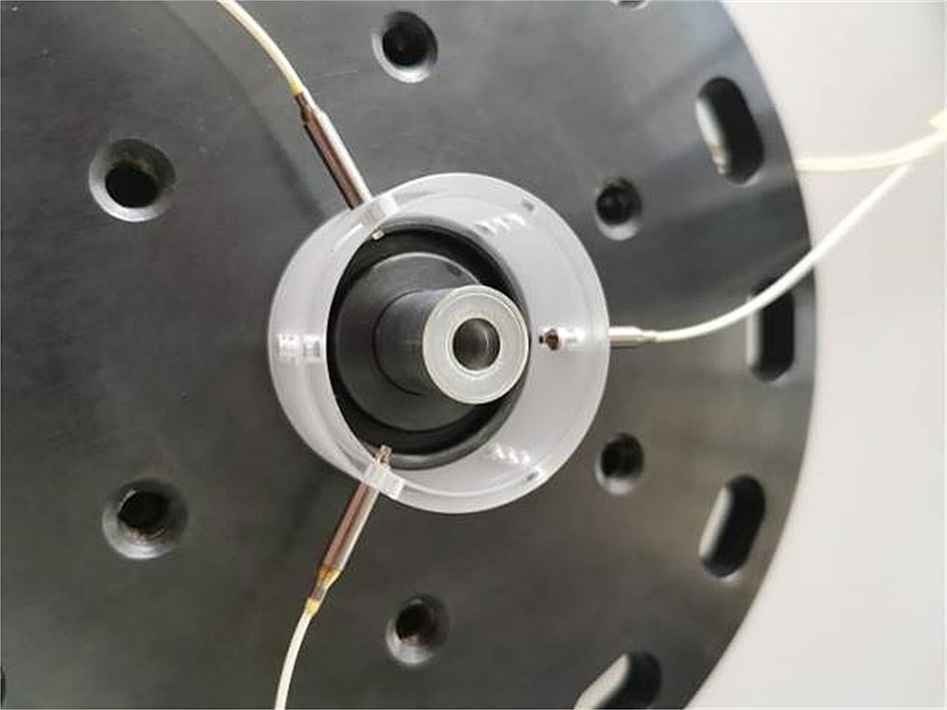
Assembled liners and external photonic Doppler velocimetry probes.

Using the diagnostic method described in reference [2], PDV probes were used to measure the velocities of the free surfaces of the inner and outer liners, denoted here as *V*_1_ (the implosion velocity) and *V*_2_ (the expansion velocity). Measurements were made for each liner using three probes, which were uniformly arranged on the circumference, such that the symmetry and stability of the liner motion could be determined by comparing the three similar PDV measurement results. *V*_1_ was measured with endoscopic multiplex (three-point) PDV probes, such that there were three measured *V*_1_ histories. The radii of the internal probes were approximately 0.68 mm, and the three multiplex probes (labeled N_1_ (0°), N_2_ (120°), and N_3_ (240°)) were arranged at intervals of 120°. Meanwhile, *V*_2_ was measured with three directly inserted single-point PDV probes (labeled W_1_ (0°), W_2_ (120°), and W_3_ (240°)) that were evenly distributed on a large circumference. The initial distance between the external probes and outer liner was approximately 4 mm. The arrays of PDV probes were located precisely at the mid-height of the liner as shown in Fig. 4. The uncertainty in the PDV measurement was less than 1%^35^. The bandwidth of the oscilloscopes for PDV signal acquisition was set at 16 GHz and the acquisition rate at 25 GS/s.

## Experimental results and analysis

**Figure 5:**
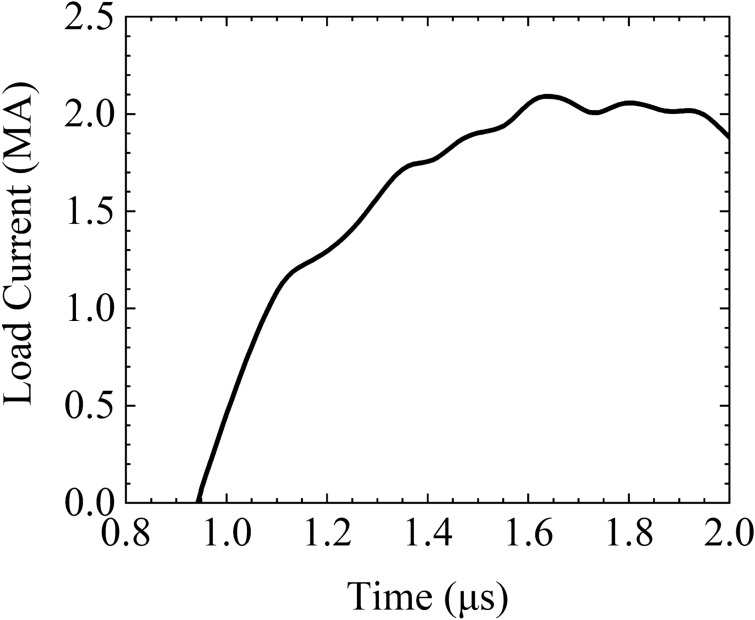
Load current waveform of CQ3-shot137.

The load current of the experiment is plotted in Fig. 5. The peak value is approximately 2.1 MA and the rise time (10–90%) is approximately 480 ns. The original current waveform was measured with the Rogowski belt mounted on the cathode flange in a complex noisy environment and with poor calibration in the mega-ampere range. Additionally, the waveform was disturbed by noise owing to the confluence of many current cables and poor electromagnetic shielding in the pulsed power experiments. The current plotted in Fig. 5 has been revised by solving the inverse problem with MHD code and optimization code to eliminate noise, according to the history of the well-measured expansion velocity of the outer liner. This approach can be considered an indirect calibration for great pulsed currents, and the revised waveform can be used as the formal load current in MHD modeling.

**Figure 6:**
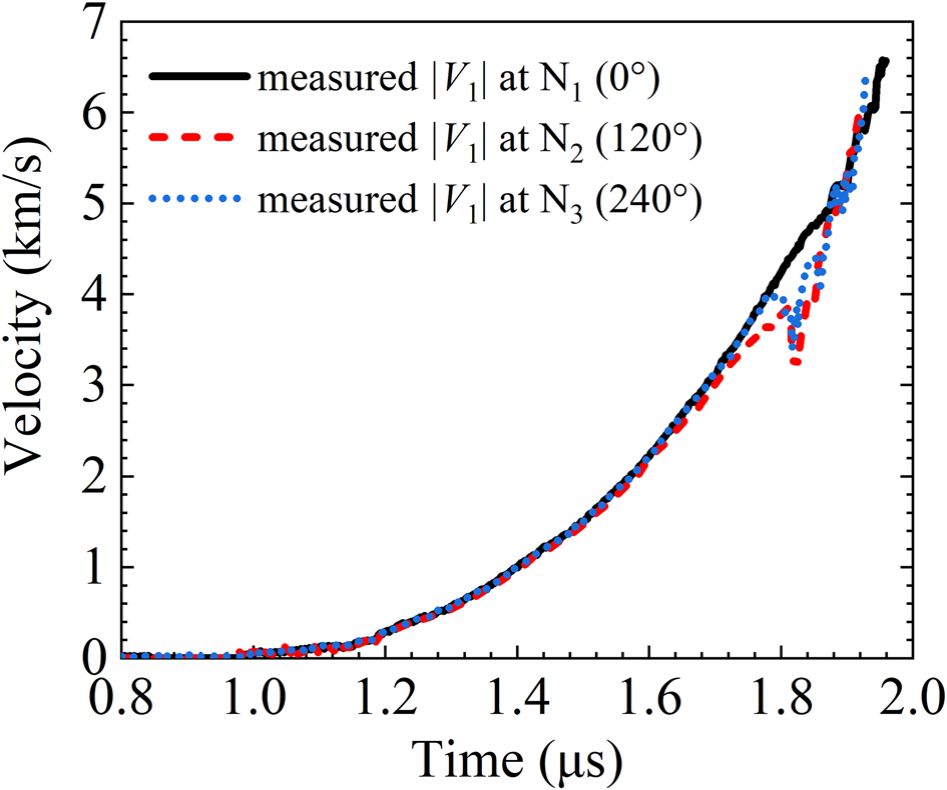
|*V*_1_| measured with the internal photonic Doppler velocimetry probes versus time.

The implosion and expansion velocities are negative and positive respectively, but for the convenience of comparison, the implosion velocities are expressed in absolute values below. All six points of the PDV probes provided reliable data. The implosion velocity |*V*_1_| curves measured with the internal PDV probes at points N_1_, N_2_, and N_3_ are plotted in Fig. 6, with the highest velocity being 6.57, 5.98, and 6.43 km/s respectively. Before |*V*_1_| reaches 2.5 km/s, the three curves almost coincide, indicating that the inner liner maintained an axially symmetrical and stable shape during that time. When the N_1_ curve reaches approximately 4 km/s, the N_2_ and N_3_ curves have decelerating fluctuations for a short period. However, the curves again coincide with the N_1_ curve as the velocities again increase and exceed 5 km/s. Approaching the peak of each |*V*_1_| curve, the N_2_ and N_3_ curves become steeper than the N_1_ curve.

The fluctuation of the velocity curves is explained as follows. (1) The implosion-driven shock wave in the air and its reflection wave within the inner liner impact the endoscopic probe rod or its micro-optical components. (2) The refractive index varies abruptly in air near the shock wavefront, and the optical path and optical interference signals then change.

The N_1_ velocity curve appears almost unaffected by any asymmetric factor. Hence, we take the N_1_ curve as the history of the normal velocity of the inner liner. The three |*V*_1_| curves are consistent over a long period and especially in the initial and final stages of the implosion. The coincidence indicates that the inner liner shape was in general symmetric and stable during the implosion.

**Figure 7:**
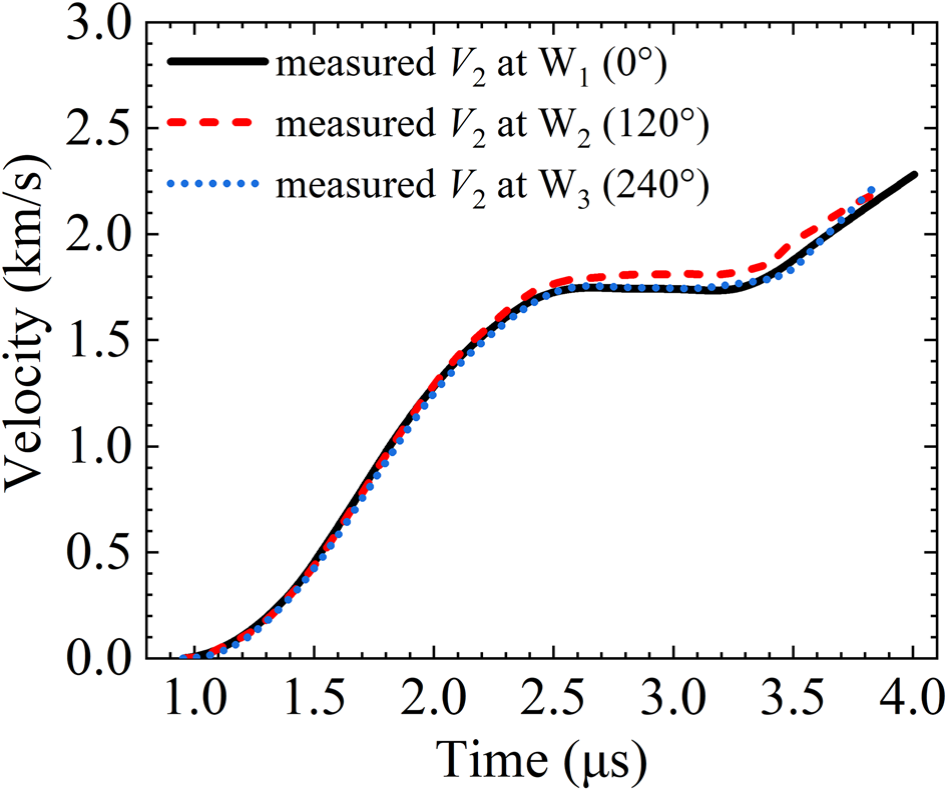
*V*_2_ measured with the external photonic Doppler velocimetry probes versus time.

Figure 7 shows the expansion velocity *V*_2_ curves obtained with the external PDV probes. The three curves coincide well, indicating that the outer liner maintained axial symmetry and stability in the expansion. When *V*_2_ reached approximately 2.2 km/s, the outer liner collided with the external PDV probes, resulting in the cutoff of PDV signals. It is worth noting that there was a microsecond period (approximately from 2.45 to 3.45 μs) in which the liner expanded at an approximately constant velocity (~ 1.8 km/s) and behaved like a freely expanding cylinder. Consequently, the magnetically driven expansion of the outer liner might be a promising loading technique in dynamic fracture and fragmentation experiments of material dynamics. This pattern can be attributed to the load current waveforms of RLC circuits generally being attenuated sinusoidal waveforms. When the load current is near the half period, the current is low and the magnetic pressure is almost zero, resulting in an almost unchanged expansion velocity. When the load current enters the negative half cycle, the magnetic pressure continues to accelerate the outer liner.

The above results clearly show that the magnetically driven liner implosion experiments take advantage of a longer steady and effective loading time, because there is a lack of transverse and azimuthal rarefactions in the mechanics and electro-magnetics. Figures 6 and 7 show that the effective loading times of the inner and outer liners were approximately 1 and 3 μs (i.e., as long as 2 and 6 times the rise time of the load current respectively) such that there was greater utilization of electromagnetic energy in the experiment.

## Discussion of liner motion and material states

### Simplified calculations using thin-shell model

It is important to make an extensive comparative analysis of liner configuration parameters and estimate main results in advance by performing a simple but reliable procedure. An analytical thin-shell model incorporating circuit equations has been used for this purpose. The model provides a theoretical basis for the load configuration design and helps clarify the basic regulation of liner implosions.

In the thin-shell model, the inner and outer liners are both simplified as thin cylindrical shells of zero thickness and with concentrated mass. The two thin shells are simultaneously driven by the electromagnetic force to implode and expand, where the dynamic inductance increases with the separation of the liners and thereby affects the load current.

In addition to adopting the thin-shell hypothesis, we make three assumptions. (1) The motion state of the liner’s effective height part is uniform and consistent, and only the characteristic radius *r* is important to the radial motion (i.e., we adopt a zero-dimensional model). (2) The mechanical work done by the magnetic pressure is completely converted into the kinetic energy of the liners. (3) The resistance of the liners is naturally far less than that of the transmission lines and thus can be ignored.

The magnetic pressure acting on the loading surfaces of the liners should be balanced with the inertial force in the positive/negative radial acceleration of the liner mass per unit area. The motion equations of the inner and outer shells are thus obtained respectively. The circuit equations are expressed with lumped parameters of the pulsed power device. The complete equations of thin-shell motion model in the SI system are

1 $$\left\{\begin{array}{c}\frac{{d}^{2}{r}_{n}}{{dt}^{2}}={\left(-1\right)}^{n}\frac{{\mu }_{0}{I}^{2}}{4\pi {M}_{n}{r}_{n}}, \left(n=1, 2\right) , \left(1a\right)\\ \frac{1}{{C}_{0}}\left({U}_{0}{C}_{0}-{\int }_{0}^{t}Idt\right)=\left({L}_{0}+L\right)\frac{dI}{dt}+I\frac{dL}{dt}+{R}_{0}I , \left(1b\right)\\ L=\frac{{\mu }_{0}l}{2\pi }\mathrm{ln}\frac{{r}_{2}}{{r}_{1}} . \left(1c\right)\end{array}\right.$$

Here, *r*_*n*_ is the characteristic radius of the *n*th thin shell; *M*_*n*_ is the mass of liner *n* per unit length, where the subscript *n* = 1, 2 respectively denote the inner and outer shells (whereas in other sections, *r*_1_ and *r*_2_ are respectively the inner radius of the inner liner and outer radius of the outer liner according to the definition in Fig. 2); μ_0_ is the vacuum permeability; *I* is the load current; *C*_0_ and *U*_0_ are respectively the capacitance and charging voltage of the device; *L*_0_ and *R*_0_ are respectively the inductance and resistance of the transmission lines, *L* is the dynamic inductance due to the deformation of the load configuration; and *l* is the effective height of the shells.

As the kinetic energy integration of the first equation of Eq. (1), it is yield that

2 $${dV}_{n}^{2}=d{\left({dr}_{n}/dt\right)}^{2}={\left(-1\right)}^{n}\frac{2{\mu }_{0}}{4\pi {M}_{n}}{I}^{2}\left(t\right)\frac{{dr}_{n}}{{r}_{n}} .$$

In general, pulsed currents reach their peak *I*_max_ about at the first quarter of cycle *T*_1/4_. Integrating Eq. (2) over interval (0, *T*_1/4_), and applying the integral mean value theorem, we can obtain an estimate to the maximum velocity of liner *n*:

3 $${V}_{n,max}={\left(\frac{{dr}_{n}}{dt}\right)}_{max}={\left(-1\right)}^{n}{\left[{\left(-1\right)}^{n+1}\frac{2{\mu }_{0}}{4\pi {M}_{n}}\mathrm{ln}\left(\frac{{r}_{n0}}{{r}_{nf}}\right)\right]}^{1/2}I\left({t}_{n}^{*}\right)$$

where the second subscript 0 or *f* represents the corresponding physical quantities at the initial time *t* = 0 or *T*_1/4_ (or at the cutoff time of implosion). $I\left({t}_{n}^{*}\right)$ is the mean value of current at the appropriate time ${t}_{n}^{*}$. Equation (3) can approximately provide the maximum implosion/expansion velocity of liners. It should be emphasized that the main physics quantity to determine the implosion velocity is the load current peak *I*_max_. Also the maximum implosion velocity is proportional to the rising rate of load current since the rise time of current for a certain devise is basically fixed. Generally speaking, the shorter rise time is required when we select pulsed power devices in order to obtain higher implosion velocity.

**Figure 8:**
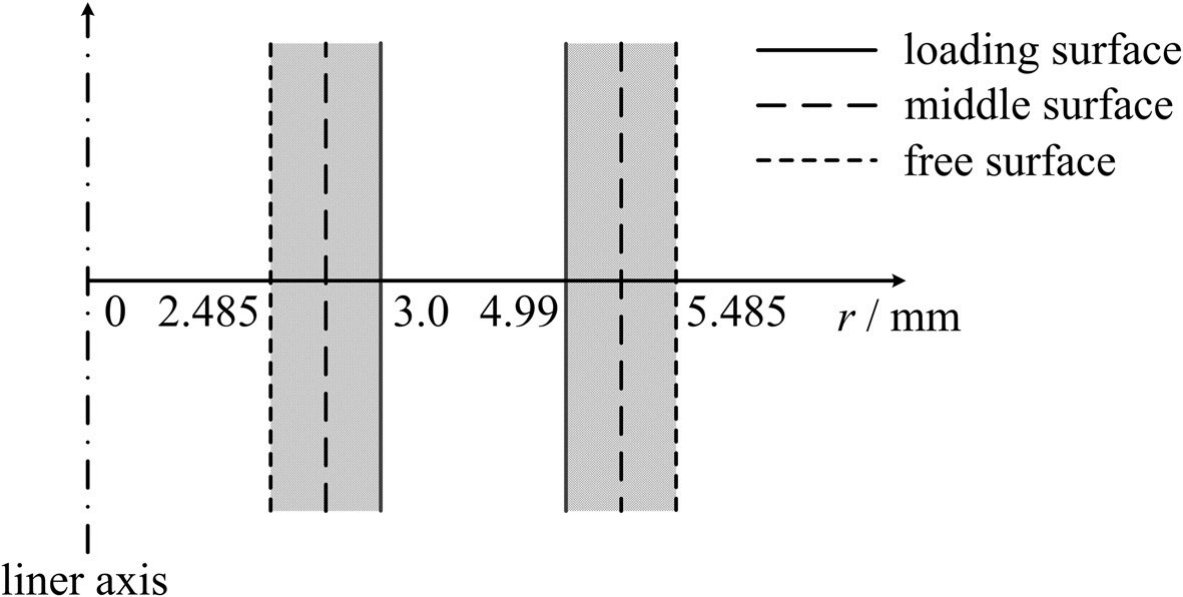
Three radial positions on a section of the inner and outer liners.

The velocity and load current histories can be obtained by solving Eq. (1) because the circuit parameters of the pulsed power device are constants (refer to Table 1). In Fig. 8, the notations of the three radial positions of the liners on their sections are plotted as the candidates of the characteristic radii in the thin-shell model. These radial positions denote the loading surface (i.e., the current flow passing surface), middle surface, and free surface of the liner.

**Table 1:** Calculation parameters of the thin-shell model.

*C*_0_	*L*_0_	*R*_0_	*U*_0_	*l*	density (ρ)	μ_0_
19.52 μF	15.76 nH	5.75 mΩ	80 kV	10 mm	2.78 g/cm^3^	4π × 10^–7^ H/m

**Figure 9:**
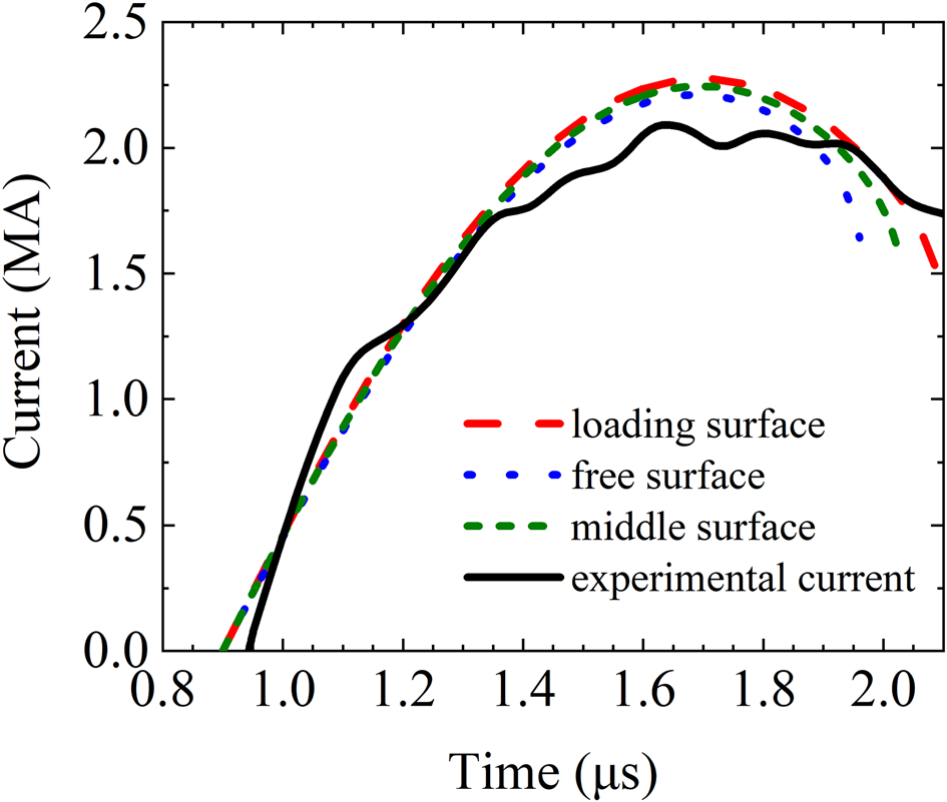
Calculated load current curves for the thin-shell model for different initial radial positions as the characteristic radius.

**Figure 10:**
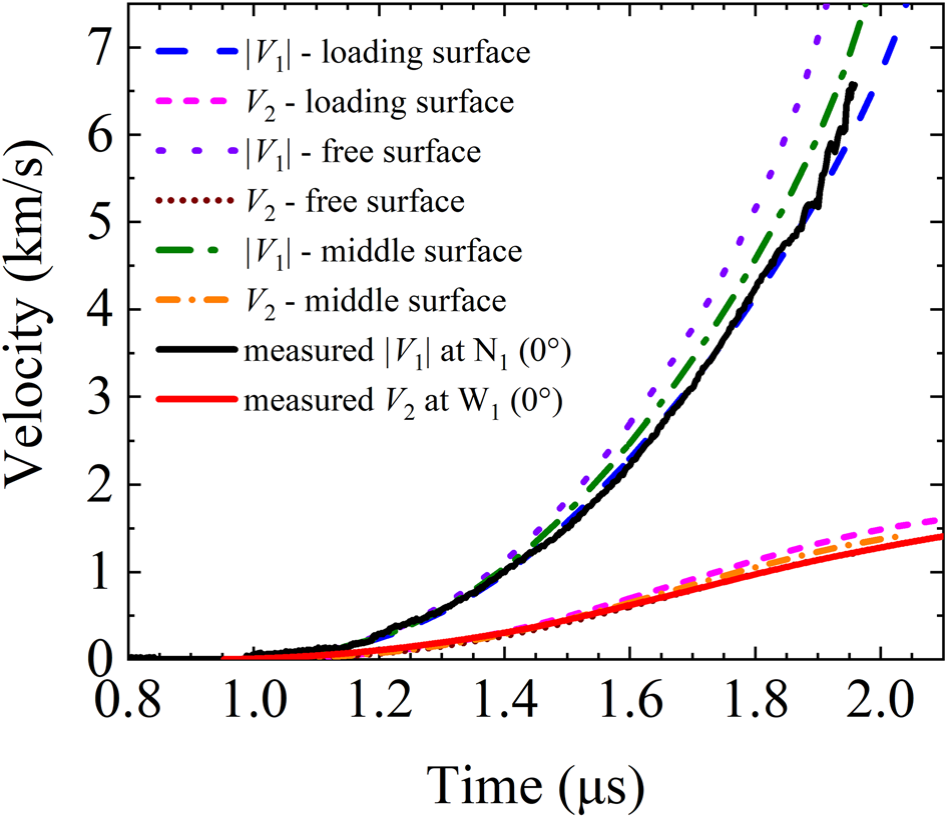
Calculated velocity *V*_*n*_ for the thin-shell model with different initial radial positions as the characteristic radius.

Taking the positions of the three radii shown in Fig. 8 as the initial characteristic radii in the thin-shell model, we get the three calculated load current curves in Fig. 9 and the three pairs of calculated thin-shell velocity curves in Fig. 10. The calculation is artificially cutoff at *r*_1_ = 0.5 mm to avoid the velocity trending to infinity as *r*_1_ approaches zero. The different choices of the initial characteristic radii would affect the calculated dynamic inductance and lead to a slight difference in the calculated current curves in Fig. 9. The peak values of the calculated load current are higher than the experimental measurements because the circuit parameters used here are obtained for the short-circuit discharge without various dissipations in that with realistic loads.

The velocity residual norm ${V}_{res,n}=\sqrt{\sum {\left({V}_{\mathrm{shell},n}-{V}_{\mathrm{exp},n}\right)}^{2}}$ is used to evaluate the agreement between the calculated and measured velocity curves, where the summation is over hundreds of sampling points. Figure 10 reveals that the best agreement is achieved by taking the loading surface radius and free surface radius as the initial characteristic radii for the inner and outer liners respectively. It also shows that the thin-shell model can effectively simulate the motion behavior of the liners and can be used in the design of physical experiments.

### Material states in the implosion process

Generally, experiments could provide velocity information, but some important physical properties of the liner materials, such as the pressure and temperature, can only be revealed by numerical simulation in which material models such as equation of state (EOS), strength and electrical resistivity are prudently chosen. A high-velocity liner implosion involves hydrodynamics and electromagnetic and plasma dynamics and thus needs to be investigated through MHD modeling.

A one-dimensional (planar, cylindrical, or spherically symmetric geometry) MHD code, SSS-MHD^36^, has been developed to simulate the magnetically driven liner implosion process. The SSS-MHD code can be used for the multi-physics calculation of complex problems in a wide range of research on liner implosion that involves hydrodynamics, electrodynamics, material dynamics, plasma physics, detonation physics, and pulsed power techniques. The SSS-MHD code is multifunctional and suited to material dynamics, detonation physics, laser effects, and multi-media and multi-area calculations besides MHD simulation incorporating real circuits and load configurations.

The MHD modeling scheme (i.e., a stationary liner configuration) is illustrated by the hatched pattern in Fig. 8, where the gap between the liners is a vacuum cavity. The HOM equation of state model^37^ and Burgess resistivity model^38^ are also used. The constitutive model used is a modified SCG model^39^.

Before performing the MHD modeling for the full load configuration, it is necessary to revise the original measured load current waveform to eliminate noise and oscillations. The revised current waveform can then be reasonably used as the real load current in the modeling. It might be thought that we can obtain a plausible load current if using a backward integration program and then input that current with a well-measured *V*_2_ curve for a long period (with the first 2.0 μs being sufficient). However, the backward integration is difficult for MHD equations because of the diffusivity of the magnetic field equation. Hence, an approach of the forward SSS-MHD simulation incorporating an optimization program is used for the revision as follows.

We input the original current waveform measured (at point W_1_) by the Rogowski belt into the SSS-MHD code to obtain the first calculated *V*_2_ curve. Then, using the optimization program to firstly revise the original measured current waveform according to the difference between the calculated and measured *V*_2_ curves, we attain the first revised load current. We then input the first revised load current into the SSS-MHD code to start the second cycle and repeat the above procedure to complete the second cycle. The iteration continues until the residual norm *V*_*res,*2_ of the calculated and measured *V*_2_ curves is less than the given limit.

**Figure 11:**
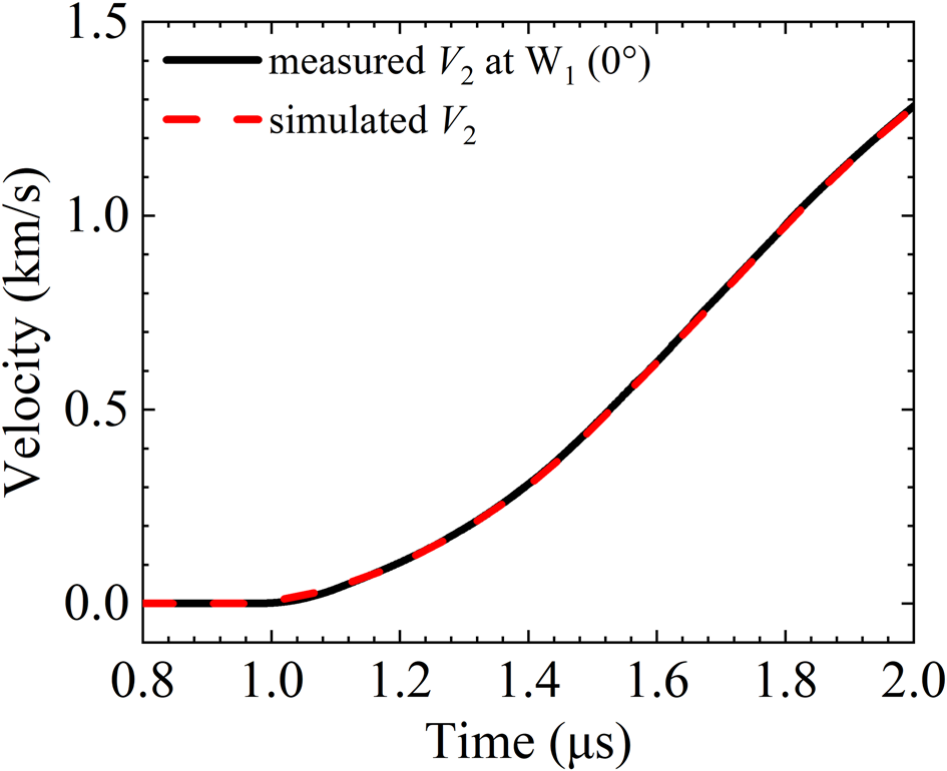
Measured and MHD simulated expansion velocity *V*_2_ curves.

**Figure 12:**
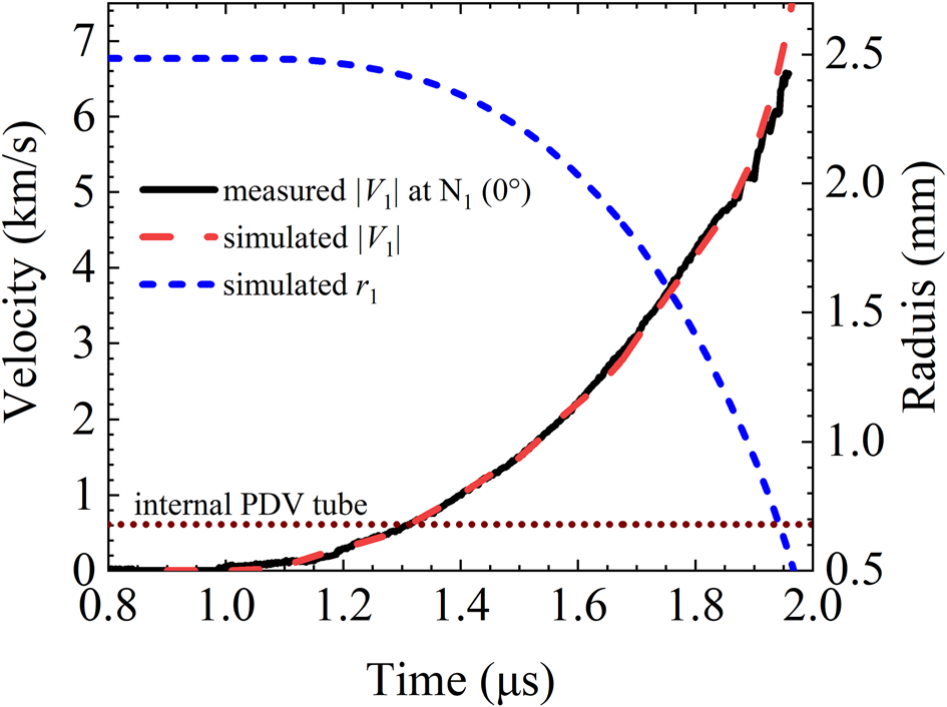
|*V*_1_| and *r*_1_ simulated with SSS-MHD. The solid black line is the measured |*V*_1_| curve at N_1_ (0°).

Taking the revised load current waveform (plotted in Fig. 5) as the loading condition, the implosion and expansion process of the two liners of the experimental configuration are simulated with SSS-MHD code. The simulated *V*_2_ curve coincides well with the measured curve as shown in Fig. 11. The simulated |*V*_1_| and *r*_1_ curves are plotted in Fig. 12. The simulated and measured velocities are in good agreement before |*V*_1_| reaches 5 km/s. Thereafter, they diverge with an average relative error less than 4.8%. The main reason for the divergence is that the EOS parameters and resistivity model used in the simulation may differ from the actual situation.

**Figure 13:**
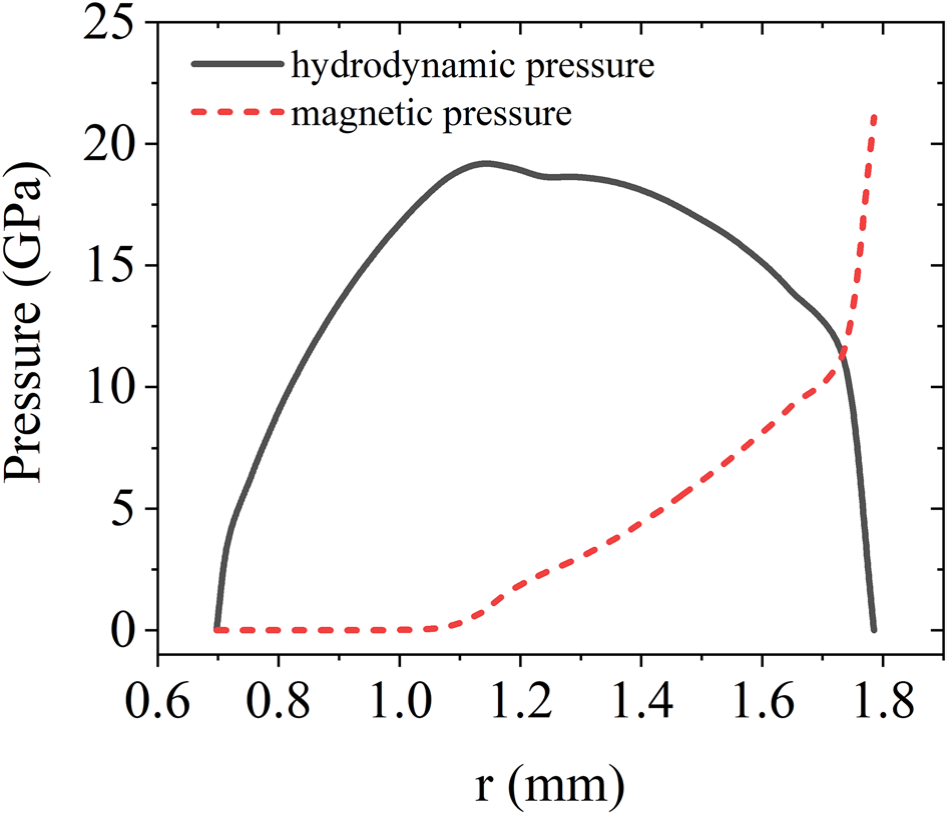
Radial distribution profiles of the hydrostatic pressure and magnetic pressure within the inner liner at a time of 1.94 μs.

Figure 13 presents the radial profiles of the hydrodynamic pressure and magnetic pressure within the inner liner at a time of 1.94 μs (when |*V*_1_| reaches 6.57 km/s) calculated with the SSS-MHD code. The hydrostatic pressure acting on 2024 aluminum under the implosion ramp loading peaks at approximately 19 GPa.

**Figure 14:**
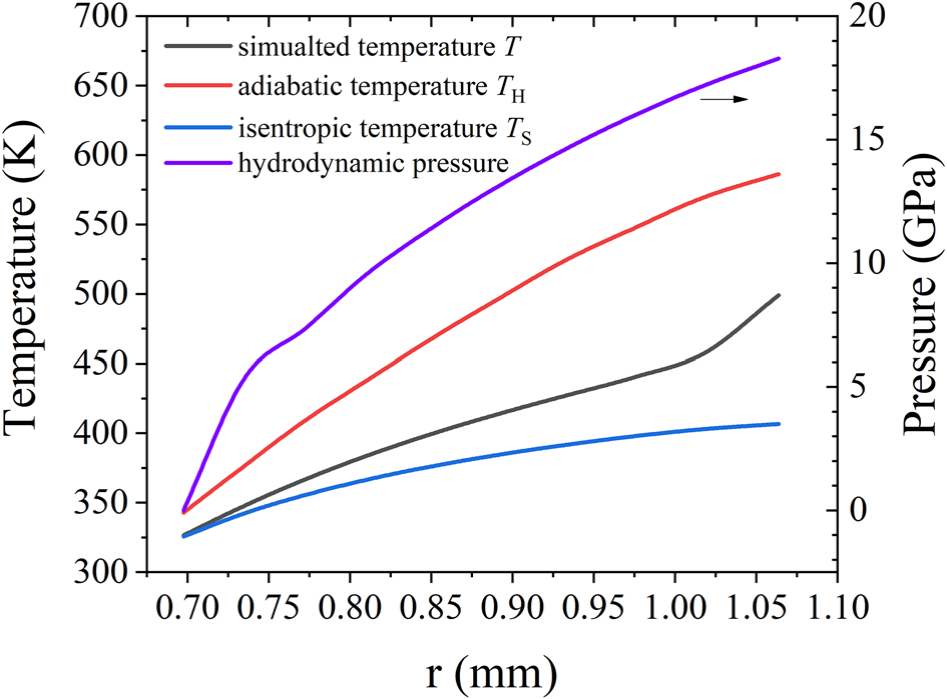
Simulated temperature, isentropic temperature, and adiabatic temperature^40^ curv es spanning the range of 0.7 mm < *r*_1_ < 1.06 mm for the inner liner at a time of 1.94 μs. The hydrostatic pressure curve at the corresponding position is also plotted.

In Fig. 13, the magnetic pressure is close to zero in the range 0.7 mm < *r*_1_ < 1.06 mm, indicating that the material is unaffected by the magnetic field and Joule heating. Figure 14 plots the simulated temperature against the radius within this range. The temperature curves of the material having undergone isentropic compression and adiabatic compression to achieve the same degree of compression at the corresponding position are also plotted. The isentropic temperature *T*_S_ is calculated using the formula

4 $$T_{{\text{S}}} = T_{0} \exp \left( { - \int_{{v_{0} }}^{v} {\frac{\Gamma }{v}{\text{d}}v} } \right){\kern 1pt} {\kern 1pt} \;,$$

where *T*_0_ is the initial temperature (300 K), Γ is the Gruneisen coefficient, *v* is the specific volume, and *v*_0_ is the initial specific volume. The simulated temperature is between the isentropic temperature curve and the adiabatic temperature curve, indicating that the material within the inner liner remains in an off-Hugoniot state during the implosion process, demonstrating that the inner liner undergoes quasi-isentropic compression instead of adiabatic compression or isentropic compression. We partially conclude that the major entropy increase is contributed by plastic deformation by employing strength model in simulation.

**Figure 15:**
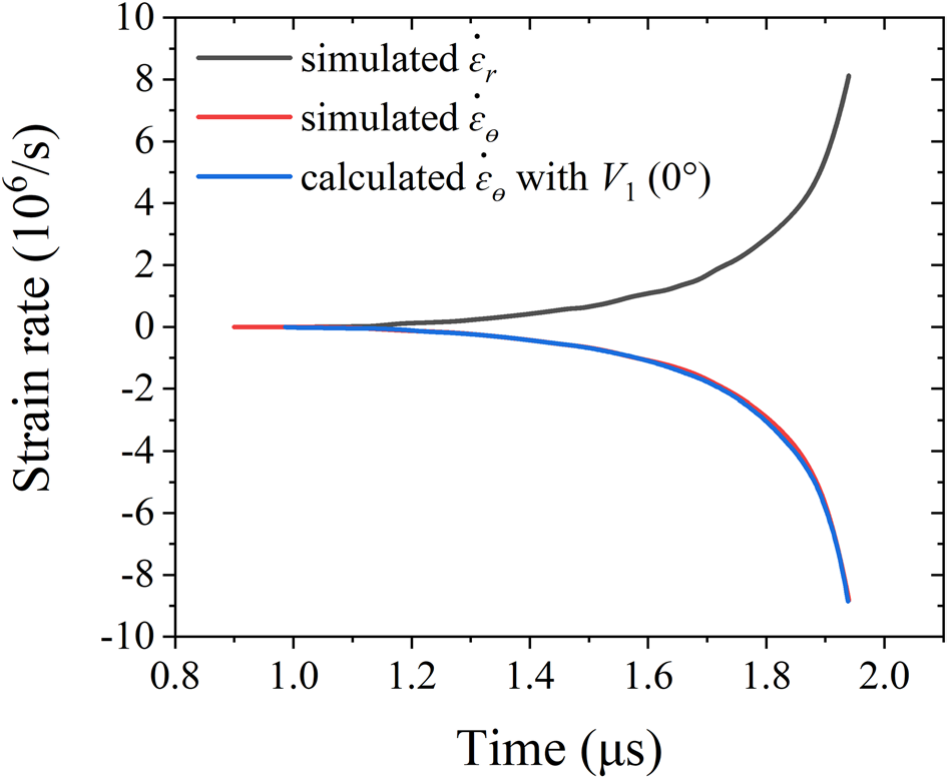
Strain rate at the free surface of the inner liner versus time.

Figure 15 plots the strain rate at the free surface of the inner liner against time in the implosion process. The curve of the circumferential strain rate calculated with the experimental measured implosion velocity *V*_1_ is consistent with the simulation result. The maximum strain rates achieved in radial and circumferential directions are 8.1 × 10^6^ s^−1^ and − 8.8 × 10^6^ s^−1^ respectively.

Confined by the diagnostics, especially the internal probe occupancy of the implosion space near the axis inside the liner, the final stage of the implosion was impossible to diagnose. It is inferred from the calculation results that it is possible for |*V*_1_| to exceed 8 km/s in the next 0.1 μs. In view of the scaling law that the maximum loading pressure is proportional to the square of the load current amplitude, it is estimated that if the load current increases to 16 MA, approximately 8 times the maximum current in this experiment, then the magnetic pressure and the hydrostatic pressure of the material within the inner liner would exceed 1 TPa (approximately 64 times the maximum pressures in this experiment), which is the experimental result obtained on the Z machine.

## Conclusion and future work

A magnetically driven metal liner high-velocity implosion experiment was successfully carried out on the compact pulsed power device CQ-3. Imploded by a pulsed load current with a peak of 2.1 MA and a rise time of 480 ns, the implosion velocity of the 2024 aluminum liner with an initial inner diameter of approximately 5 mm and a thickness of approximately 0.5 mm reached 6.57 km/s (before the implosion cutoff). The outer liner as the current returning channel, as well as a comparison sample, accelerated continuously, and its velocity exceeded 2 km/s before the liner impacted the external probes. The shapes of the liners remained axisymmetric and stable during the measuring time of the experiment (approximately 1 μs for the implosion and 3 μs for the expansion).

The thin-shell model can be used to simulate the dynamic behaviors of liners effectively and are suitable for the design of the physics of experiments. Using SSS-MHD code and the optimization program, the measured load current is revised with reference to the well-measured expansion velocity. The revised load current is in turn used as the loading condition in the overall MHD simulation of the full configuration. The history of the simulated implosion velocity agreed well with that of the measured velocity. At the same time, the radial hydrostatic pressure profiles of the 2024 aluminum of the inner liner at different times were presented, with the maximum pressure being approximately 19 GPa. It is expected that quasi-isentropic compressions of metals up to hundreds of GPa will be obtained when accelerating the inner liners to implosion velocities of 10–20 km/s using compact high-quality devices with higher load currents.

In future work, the current transmission and diagnostic techniques need to be improved further. The oscillations on the load current should be minimized, and the full implosion process of the inner liners should be improved. There is a need to explore further the processing of implosion experiment data and related theories and a better load configuration design, to obtain high-precision and self-consistent pressure and EOS data. This article and related future work might provide a basis for experimental exploration and data processing in research on the magnetically driven implosion of metal liners experiencing ultra-high pressure and compression.

There remain many innovative concept experiments to explore for compact pulse power devices. As an example, shaped inner liners can be used to create high-velocity macroscopic metal plasma jets traveling at tens of kilometers per second.

## Data Availability

No additional data (other than those presented in the manuscript) were produced or used for the preparation of the manuscript.
